# ICT to Promote Well-Being within Families

**DOI:** 10.3390/s18092760

**Published:** 2018-08-22

**Authors:** Jure Trilar, Andrej Kos, Simona Jazbinšek, Lea Jensterle, Emilija Stojmenova Duh

**Affiliations:** 1 ICT Department, Faculty of Electrical Engineering, University of Ljubljana, Tržaška c. 25, 1000 Ljubljana, Slovenia; andrej.kos@ltfe.org (A.K.); simona.jazbinsek@ltfe.org (S.J.); emilija.stojmenova@ltfe.org (E.S.D.); 2 Faculty of Sport, University of Ljubljana, Gortanova 22, 1000 Ljubljana, Slovenia; leajen@gmail.com

**Keywords:** ICT, sensors, well-being, family-centered design, healthy lifestyle, family time

## Abstract

Within the Active Living and Well-Being Project (RRP3), funded by the Republic of Slovenia and the European Regional Development Fund Investing in Your Future program, we aim to develop different approaches and prototype solutions to provide ICT solutions for the family in order to connect its members; communicate; promote quality family time, active life, a health-friendly lifestyle and well-being; and integrate various sensor and user-based data sources into a smart city ecosystem platform. A mixed methodology, combined qualitative and quantitative approaches, was selected to conduct the study. An online survey with a structured questionnaire as well as semi-structured interviews were performed. Through the analysis of the results, we tried to establish a family-centered design approach that would be inclusive as much as possible, creating benefits for all generations in order to develop an interactive prototype solution that would allow us to further test and verify different use-case scenarios.

## 1. Introduction

Advances in ICT, the advent of the Internet of Things, and an exponential increase in the number of ICT users, smart sensors and IoT devices, connections and interactions established between multiple human and non-human agents have made possible the emergence of new technology usage patterns. They also require constant learning of new skills.

Recent attitudes towards everyday use of ICT were presented in March 2017, by Eurobarometer (460) in the *Attitudes Towards the Impact of Digitisation and Automation on Daily Life* report, showing the impact of digital technologies and the way European citizens perceive digital transformation. A majority of the survey respondents were very positive about the impact the most recent digital technologies have had on the society, the economy and their quality of life [1].

However, it can often be difficult for individuals connected in the interpersonal dynamics of various social structures to adjust to the evolution in everyday ICT usage. This could be demanding in terms of general well-being, health and healthy long-lasting relationships. With regard to the particular position in the contemporary world, families are frequently burdened with excessive use of technology by some members, commonly the younger generation, and inadequate usage of convenient ICT solutions by others, mostly the elderly. To tackle communication and other challenges within the family, we aim to develop an innovative ICT solution that would enable better communication and organization in order to relieve stress, provide more gratifying time for socializing, and promote an active and healthy lifestyle among family members of different generations.

In this article, we focus on establishing a methodology for a coherent approach towards designing and developing (digital) solutions following the requirements of a family in the modern, interconnected world. Initially, we present familiar concepts regarding the use of ICT in everyday family life, the organization of family life, time management and a healthy lifestyle. Then, in the consecutive chapter, we disclose motives regarding the EkoSmart project use-case. Next, we announce an ambition for a broader methodology examination, as we propose an extension to established aspects of user centered design approaches—a family-centered design.

The study is explained in the following chapter and covers the demographic structure of study participants, how they were selected, and how the survey and interviews were conducted. A detailed analysis of the results and discussion regarding the key aspects, as well as the limitations of the study, are presented in the penultimate chapter. We conclude with an overview of key topics and findings covered in this paper.

## 2. Background

In this section, we explore routine practices of family members and their impact on family organization and time management in order to discover potential benefits on well-being through the use of ICT solutions in everyday family life. The literature review and state-of-the-art analysis helped us introduce a basis for an online survey and semi-structured interview implementation for further examination and prototype solution development.

### 2.1. The Use of ICT in Everyday Family Life

ICT has an effect on family functioning, creating new interaction scenarios and rearranging current family relational patterns [2]. Difficulty in establishing patterns of ICT use is even broader than in single-user scenarios. Van Rompaey, Roe and Struys [3] created a typology of family ICT possession: The traditional type is characterized by low technology density, intermediate density (includes more televisions and audio systems), and multimedia family type (possesses a greater amount of new technologies, e.g., the Internet and email).

Furthermore, the perceived dichotomy between “explorative family” and “traditional family” communication strategies was noted by an Ericsson consumer lab report in 2016 [4]. Explorative families are frequent users of novelty means of communication channels and more plural media types, and are more prone to try out new services, whereas traditional families mainly use established means of communication, e.g., mobile text and voice, among their family members. A similar notion of explorative families in the form of multimedia families was characterized by the possession of new technologies as part of a high technological density [3]. Parents in the explorative family type tend to have a higher knowledge of communication services, thus having greater control and ability to monitor their children’s engagement through ICT.

We must take into consideration not only the use of technology but also how it is being used and what it is being used for. Thus, the importance of parents following up on their child’s unsupervised media interaction by engaging in conversation should also be noted. Unintended effects could be damaging to the manner in which parents communicate with their children (via ICT) and possibly hinder the development of their family relationships. Different technology use could often have at least two outcomes: To serve as a medium for independent user activities or as a vessel for socializing and communication [5].

Some studies suggest that ICT is becoming a focal point in the various stages of the family life cycle, with the individuals and families adopting these technologies not only in regard of functionality and features, but also the families’ own characteristics (e.g., socioeconomic status, cultural differences, geographic location and distance between family members, household type and communication strategies) and development stage, thus differently impacting family functioning in accordance with the specific phase of the family life cycle [2].

Various authors suggest that ICT is blurring boundaries between work and home [6], affecting social interactions and family cohesion by increasing privatization within family life [7], reducing interactions [8], increasing the digital gap between generations, redefining family roles, producing intergenerational conflicts [9], and therefore lowering the quality of family relationships. On the other hand, ICT can positively affect family dynamics by creating a shared experience [9,10], i.e., when teaching another how to use technology [10], enhancing feelings of safety [11], increasing the time families spend together, improving communication and connectedness between family members [12,13] and helping to maintain family relations [2].

### 2.2. Organization of Family Life and Time Management

Domesticated ICT provides new benefits for a household and family, and has changed the understanding of common family time. Such processes are not new; at least since 1985, the notion exists that the allocation of time and technology use patterns have changed the organizational logistics of shared family time in the case of introduction of television and desktop computers. New technologies have created new use options for family members by “enhancing different patterns of social interaction, access to information and allocation of time” [14]. For example, one way that ICT positively influences social interaction among family members is that they reduce “the effort required to perform tasks- and work-related activities, and thus allows households to engage in non-task activities” [14]. In this manner, leisure time is increased, thus providing individuals with more optimization in regard to how their free time is spent. ICT allows “additional control over one’s life” and can thus increase social interaction [14].

Several studies analyze the effects of such family rituals on family cohesion, its identity, and particularly children’s well-being, and demonstrate that they can have positive impacts on all these aspects [15,16,17,18]. In addition, several of our participants also remarked that routine mealtimes throughout the course of the week serve as sites for socialization and transfer of knowledge about healthy nutrition and eating habits, which is seen as an important aspect, especially in families with young children [19,20].

### 2.3. Healthy Lifestyle

The perception of health and a healthy lifestyle is subject to different meanings to different people. Åstedt-Kurk et al. [21] noted that a general sense of well-being, including components such as harmony, balance, and satisfaction, is an integral part of the individual’s experience of good health. An important aspect of a general notion of an individual’s health is also the sense of life control, i.e., the ability to lead an independent life and to decide on the use of one’s resources. ICT tools in the form of progressive web applications or web services, often augmented with smart sensors, as a shared, connected experience among family members offer a somewhat natural fit to analyze an individual’s and family’s health performance over lengthy periods, and provide logistics management regarding common family, health-related or other activities.

The use of ICT in home care or eHealth is an expanding research area with a variety of applications. The result shows that ICT in home care is mostly used as a tool for communication between healthcare professionals and patients or family members [22]. Despite a prevalent positive attitude towards the use of eHealth applications, ICT cannot replace a face-to-face encounter, but can be used as a complement [22].

The Internet is also a venue for social interactions and may be a means of social support for patients [23]; in our case, elderly family members. Using eHealth applications may provide an opportunity to exchange information and promote a sense of support among group participants. However, given that virtual communication is concerned primarily with the production and maintenance of bridging social capital or “weak ties”, virtual communities have limitations in terms of the social support they can facilitate [23]. In this regard, we propose the swap of “weak ties” with “family ties”, which are “strong” by definition, in order to facilitate strong social support via ICT applications for managing family health activities. According to Lindsay in 2008 [23], “ICT may by a ‘highway to health improvement’ for some aspects such as diet, and may work for some people, but we should not underestimate the amount of time and resources it takes to help people overcome barriers in learning how to use a computer.”

Health and care services are in demand, as 52% of Eurobarometer’s respondents in March 2017 would like online access to their medical and health records. It is also important to note concerns regarding cyber security and privacy issues in the potential use of medical data for measuring everyday activity, as the average users are much more willing to share their health and wellbeing data with doctors and healthcare professionals than with public authorities, public sector companies, or private sector companies—even if anonymized—and for research purposes [24]

## 3. EkoSmart: Active Life and Well-Being Project

The Faculty of Electrical Engineering, University of Ljubljana, together with multiple Slovenian private and public entities is involved in EkoSmart—a program funded by the Republic of Slovenia and the European Regional Development Fund Investing in Your Future program. The EkoSmart program consists of solutions in six different projects that integrate the various solutions into a common smart city ecosystem and focus on three key domains of a city for the future: Health, active lifestyles and mobility.

One of these projects is the Active Living and Well-Being Project (RRP3), in which we aim to develop approaches and prototype solutions to provide ICT support for the family within EkoSmart in order to connect its members, communicate, promote quality family time, active life, health-friendly lifestyle and well-being. In the RRP3 project, we recognize the need to achieve this purpose with solutions for the active life and well-being of the family as a model of a lasting and intimate community, in the form of MyFamily application.

**Figure A1:**
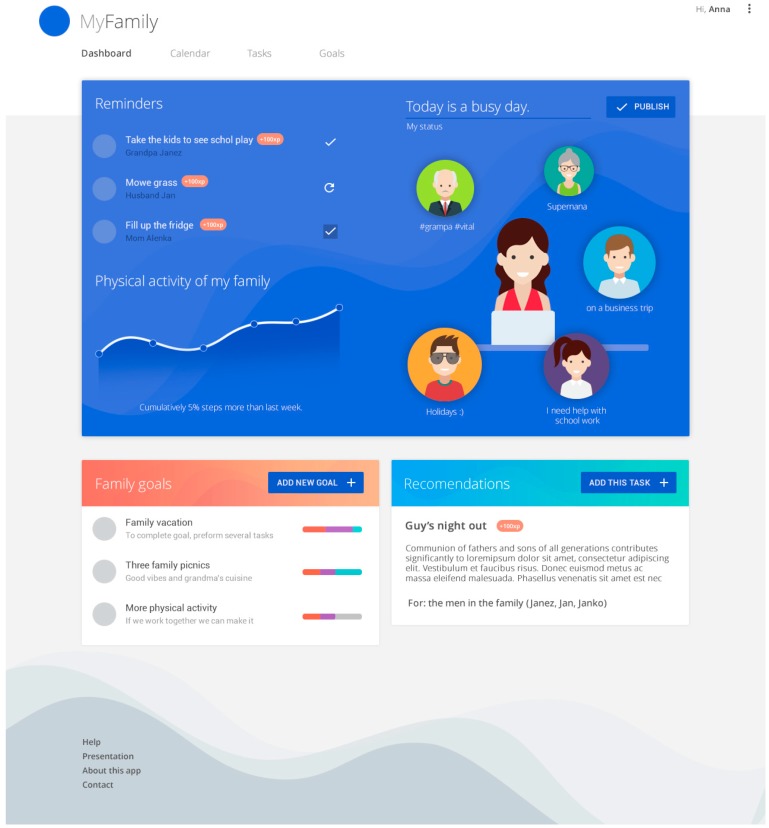
User interface of an interactive prototype solution translated into English.

**Figure A2:**
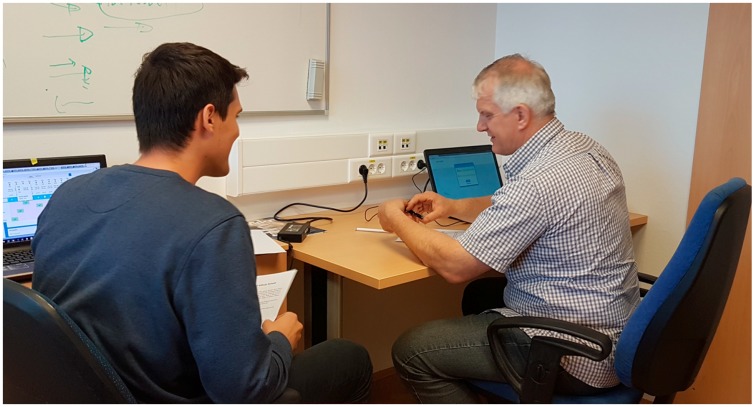
User experience testing and survey.

The key goals of the project are comprised of several tasks: Analyze user needs, describe the concept of active and healthy family in a smart city, determine motivational mechanisms for the promotion of active family life in a smart city, produce a functional specification for a solution according to the selected use-case, develop and test an interactive MyFamily application prototype (Figure A1 and Figure A2) that supports integration of external solutions and sensors via the middle data communication layer of a smart city platform.

Smart city scenarios extend the scope of possible solutions to the IoT paradigm, which would allow additional scalability and customized features of the system [25]. Furthermore, with the addition of somewhat social mechanisms of interaction among the devices, the Social IoT approach would allow for the enhancement of data trustworthiness [26].

## 4. Family-Centered Design

In an article about reframing dichotomies in human experiential design of healthcare technologies, the author Kei Hoshi explores, among other themes, the human-user dichotomy [27]. Dichotomy is projected in a dimension from customer-, user- to person- and human-centered design in a form of various parameters, from simple to more complex interactions, from less to more involving experiences, from short to longer-lasting experiences, from an isolated to a shared environment, from value-added to emotional transactions, from practical to experiential, etc. All these different (consumer-, user-, person-, human-) centered approaches have established methodologies on how to design processes and products specific to the constraints by which end-users are characterized. For illustration, the customer-centered design approach is focused on developing processes that enable the fastest, frictionless transactions of value from the consumer to service or product providers, whereas the user-centered design approach strives for optimal ways how users can, want, or need to use the product. The person-centered design takes into account more demographic and statistical data to provide a better experience tailored to each person. The human-centered design approach aims to improve user experience even further by utilizing the advances in multidisciplinary fields, considering human dignity, access and ability, while incorporating “culturally sound, human-informed, and appropriate solutions to problems in a variety of fields rather than solely product- and technology-based fields” [28].

This notion creates an opportunity for an enquiry about a possible design approach that focuses on a holistic human experience in process and product conception in the context of long-lasting meaningful relationships and frequent interactions that have an inherent impact on an individual’s behavior, with specific needs and limitations often not addressed by human-centered design approaches. For the lack of a better definition, we attempt to produce our own. Although the subject matter is broad by definition, we start with a basic construct that will allow us to expand the topic in the future. This design approach should address not only the particular individual’s needs, life situation, access and skills, but also the nature of interactions with the closest peers that influence this individual’s life in a longer-lasting common experience—typically one’s family. This design approach establishes the bottom margin extent of a group’s needs as an absolute priority, and we believe it is applicable in multiple fields of product and process development. When developing an ICT solution, this approach should entail:

- Analysis about common modes of communication
- Regard to access to technology among group members
- Shared purpose in everyday tasks
- Concern that the processes in the group should be inclusive as much as possible in order to create a better experience for all.

For illustration: In our use case, we outlined the functionalities of the MyFamily web application, minding distinct skills and access to technology of different generations. In this manner, although grandparents are typically unskilled in managing progressive web applications, they can still participate in family time organization and logistics via text messaging service for basic cell phones; and skilled users, parents and children, have a good experience using a web application via smartphones or desktop computers. Although different user interfaces are used for different generations, they all share common processes and purposes. Family-centered design takes into account the needs and requirements of all types of users involved.

## 5. Study

### 5.1. Participants

The study was conducted with family members across Slovenia. Although the term family is constantly used in everyday speech, there is no easy and universal definition. Its meaning is socially constructed and is “a matter of collective definition and human agreement” [29]. When we speak of a family in this paper, we acknowledge the complexity and pluralization of family forms and structures. We understand that many definitions and concepts, such as that of a nuclear family, fail to capture the diversity of families across and within different societies. For the purposes of our research, we opted for a broader definition and decided to refer to the notion of multi-generational families in which different generations establish and maintain important relationships, although they may not live in the same household or home. The study set out to explore how ICT is involved in their everyday lives and how it fits with people’s attitudes and current day-to-day practices. Based on this, we considered the ICT potential to promote well-being within such families.

According to the Slovenian Statistical Office [30], the most common type of cohabitation in Slovenia is a one-family household, since two-thirds of the population lived in such households in 2015. However, there were also many other types of domestic establishments. One-tenth of the population lived in multi-family households. A large majority of such households consisted of three successive generations (nine out of ten households), and more than half of their members were children and grandchildren. In addition, a smaller share (8.5%) lived in extended family households where in most cases three generations resided together. Our research extended the scope of these data, since it also took into account relationships between members of different generations that do not necessarily live together, but who perceive relations with non-residential family members as a meaningful component of their family network. 

### 5.2. Methodology

We decided on a mixed methodology, and combined qualitative and quantitative approaches. An online survey with a structured questionnaire as well as semi-structured interviews were conducted.

The online questionnaire consisted of 32 questions in 8 sections, in which the respondents assessed or reported about demography, size and form of their household, distribution of household work, family time and goals, health and care for other family members, time management and the use of ICT. Most questions (other than those on demographics) measured attitudes towards certain aspects of family life and ICT use. For this reason, a five-point Likert scale was used to indicate the degree of agreement or disagreement (1 meaning »strongly disagree« to 5 »strongly agree«) or perceived frequency of events, also on a scale of 1 to 5 (1 meaning »never« and 5 »very often«).

The participation in the online questionnaire did not predict any required conditions of the participants, but the qualitative stage focused on those participants who lived in at least two-generational households and had connections with the third generation, namely grandparents. Semi-structured interviews were chosen as a research method, as they allow participants to talk more openly and freely and to describe experiences in their own words. This generates valuable knowledge, a deeper understanding, and potentially new insights that can hardly be predicted in advance.

In-depth interviews with 16 different family members were conducted. Their aim was to examine and understand the participants’ beliefs, norms, values and behaviors regarding family lifestyle and well-being, with the emphasis on shared family time and family communication. For this purpose, we designed a list of descriptive/open-ended questions that served only as guidelines, as we adapted the course of the interview according to participants’ answers and responses. Such questions encouraged people to describe social situations in their daily lives and allowed us to learn what is important to their family members [31]. The questions focused around two basic topics: (i) Family members’ lifestyle with the emphasis on a healthy lifestyle and well-being, and (ii) family life regarding the time that families spend together. Important aspects of the discussion were how participants use ICT in their day-to-day lives to communicate with other family members and what the role of technology is in the promotion or maintenance of a healthy lifestyle. 

Interviews were audio recorded and transcribed for the analysis. All individuals participated voluntarily and signed a consent form, and their anonymity was provided throughout the research.

## 6. Results and Discussion

### 6.1. Participants

In February and May 2017, 101 respondents participated in an online survey. They were invited via e-mail and the sample was non-probable in the form of a judgement sample.

**Figure 1:**
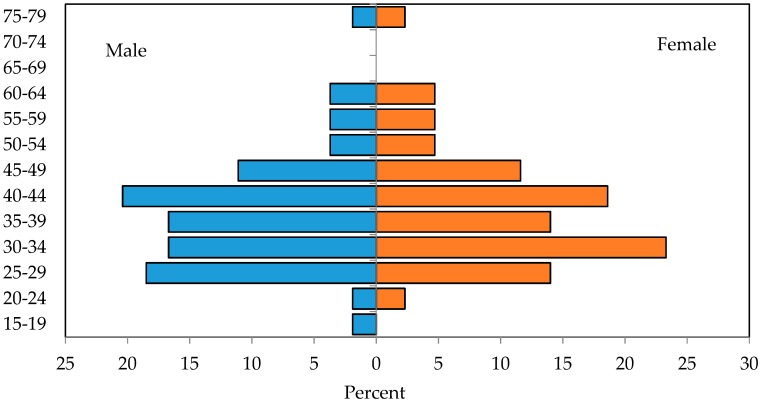
Relative distribution of survey participants grouped by gender and age.

Most of the survey respondents were parents (71%), a smaller portion were children (27%), while there were only a few grandparents (2%). The median age of all participants was 39.4 years, 39.2 median age for male and 39.7 median age for female participants. In the subsequent chart (Figure 1), distribution of survey participants grouped by gender and age is presented in an age pyramid.

Most of them were employed or self-employed (90%), while the rest were students (6%), retired (3%), or unemployed (1%). The majority lived in a one-family household consisting of four family members, namely two parents and two children. Grandparents usually lived outside, in a separate household, while in one-fifth of the cases grandparents or other family members lived together with the nuclear family. 

In March and April 2016, interviews were conducted with 11 women and 5 men. The majority of them were parents, while 3 interviews were performed with children living with their parents. The median participant age of parents was 34.5 years. Nine participants lived in two-generational families, while 7 lived in the same house with the third generation. We deliberately chose the term ‘house’, because in most of the cases mentioned, the oldest generation (grandparents or great-grandparents) formally formed their own household, despite living in the same building as their descendants. The term ‘household’ was not used because it is characterized by a socio-economic relationship between the members. Despite a limited number of participants, their family structures and stages of family cycles were very diverse—nuclear families, extended families, reorganized families, one-parent families, families with pre-school children, those with adolescents, and families with adult children living at home. 

According to the typology addressed in the Background section of this article, based on the family’s possession of ICT [3], all interview participants represented explorative (multimedia) families, characterized by the possession of new technologies as part of a high-technological density. They all owned smartphones and were daily internet users. Such families were chosen for interviews because we wanted to investigate the role of ICT in the day-to-day life of family members, and its potential to promote family well-being.

### 6.2. Outcomes

From the survey and interview analysis, the three most important themes were presented: The use of ICT in everyday family life, the organization of family life and time management, and a healthy lifestyle.

#### 6.2.1. The Use of ICT in Everyday Family Life

In 2016, 78% of all Slovenian households had internet access at home, compared to 54% 10 years before. In addition, 54% of individuals aged 16 to 74 used mobile devices to access the internet on the move and 64% were frequent users of the internet, i.e., using it every day or almost every day (Eurostat). This rapid expansion of ICT use in recent years affected various aspects of people’s everyday lives, and numerous studies on the relationship between ICT use and families were stimulated. The review of such research demonstrates changes in family functioning, but there is an ongoing debate between scholars on the effects that ICT has on families and their dynamics [2].

To understand how ICT influences family life and what leads to such diverse outcomes, we were interested in how family members use different media devices and what for. The survey analysis shows that smartphones are a device most often used for communication between family members by parents as well as children, and regular mobile phones by grandparents. 

Table 1 demonstrates that mothers mostly use smartphones (80.4%) and computers (81.4%). The results are similar for fathers, who use smartphones (83.2%) and computers (83.2%), and children—smartphones (80.2%) and computers (70.4%). However, there are generational differences, since grandmothers and grandfathers use these devices to a much-lesser extent. While only around 30% of them use smartphones, regular mobile phones are used by more than 60%.

On the basis of data analysis regarding device type usage and family-centered design approach, we propose a feature in the outlined ICT solution that would enable the inclusion of users in the form of different modalities for different devices. In this manner, the unskilled elderly would still be able to participate in family organizational processes by receiving task reminders via text messaging services (SMS) on their regular mobile phones, whereas younger family members would use enhanced interface on their smartphones or personal computers.

These devices are perceived by all participants as an indispensable means of communication, and according to the results of our study, ICT can play an important role in families, especially in relationships between family members when being apart and with extended family members, such as non-residential relatives. Most families claim it makes it possible for them to have regular contact with one another, increases communication and provides important information, such as for children’s safety. In doing so, technology has the potential to reduce geographic distances and surpass national borders, even at no extra cost. For this reason, newer internet services such as WhatsApp, Viber, Skype, etc. are commonly used. The other advantage is the ability to share photographs and videos, for example, of growing grandchildren. About that, one of the participants commented: “If we didn’t see each other for two weeks, my mum would complain she didn’t know how her granddaughter was growing up. For this reason, we started using Snapchat. We record our daughter and send her pictures and videos. She is not complaining anymore.” (Father, 35 years)

Nevertheless, phone calls, text messaging or even applications that allow video transmission and bring the experience closer to in-person communication, usually serve only as its substitute and are used primarily to fill gaps between face-to-face conversations. As one father remarked: “Technology comes in where there is a lack of socializing, when there is no time or possibility to be with a person. In fact, it is a substitute for socializing.” (Father, 35 years).

Throughout the research about the use of ICT in day-to-day family lives, it has been observed that family members decide on which service to use according to the purpose of communication. Our participants prefer phone calls mostly to organize practicalities and logistics or to deal with issues requiring immediate response. Sometimes they choose it over other media if they find it less time-consuming (especially texting seems to be less appropriate for some people or occasions). Text messages could also be used for similar purposes, but more often when sharing information that is hard to remember or that should be available for a longer time so the receiver can look at it later on, such as a grocery list. Other reasons refer to the content or context of the message; texting is preferred when people want to share information, but do not anticipate (an immediate) response, e.g., when they share interests, photographs etc., or when conversation is not possible (e.g., being at a workplace).

Positive effects recognized by individuals are often the cause and motivation for people to learn how to use different communication tools. There are differences between family members regarding skills and knowledge needed to master digital technology, and there is very often a generational divide. However, this gap can also serve as an incentive to gain new knowledge, often in a shared experience when teaching another how to use technology. The following comment supports this: “My son and his great-grandfather were very attached to each other. After we moved, we started using Skype to keep in touch. We had to visit him and show him how it works. Now he uses it and calls us because he knows it is the only way for us to see and hear each other.” (Mum, 28 years).

This knowledge allows individuals to communicate with relatives, but it also helps parents to learn virtual worlds, which are an important part of the social interactions with their children, while simultaneously allowing them to control their activities. A mother of two adolescent children remarked: “My son’s Facebook profile was stolen once, so he is more careful now. But my daughter has several accounts and is sharing a lot of information. I decided she can only use Facebook if she adds me as a friend. That way I can monitor what she shares and I try to talk with her how to use social media responsibly.” The parents of growing children that expressed the most concerns and worries about potential risks associated with ICT use, can use it as a punishment tool, pose restrictions on its use, or make different rules. The age when a child gets a mobile phone or computer access also seems to be one of the serious dilemmas they deal with differently. “We have some rules and we often confiscate our daughter’s [11 years old] phone as a punishment. We want to know when she uses it and what for. And the time she spends doing other things, such as reading or being outside in the nature, has to be twice of that she spends being on the phone.” (Mother, 32 years).

The participants reported similar negative impacts also noted in the article’s Background section, specifically examining the influence of home Internet access on children [7,8,9]. Some restrictions directly address the negative impacts of different devices on family time, perceived by participants, especially as a result of their excessive use during family occasions. Several participants in our study admitted how ICT can negatively influence communication and works as a distraction when family members are spending time together. For this reason, smartphones are sometimes not allowed during shared activities, such as meal time.

#### 6.2.2. Organization of Family Life and Time Management

Participants emphasized the importance of the nature of time spent together. The amount of time also mattered and many parents felt they would like to have more time for family, especially in relation to the time spent working and everyday commuting. However, feelings about shared family time and particularly time with children were more associated with the structure than the quantity of family time. Shared activities within and outside of the home were regarded as most significant for the quality of family functioning. One mother described: “We go for a walk, up to the hills, on the playground, in the park, to museums, zoo. Even if we go to grandma’s over the weekend, we do not stay in the house, but we rather go somewhere to do something together/.../we make experiments, plant flowers, run wild, bake cakes, cook, paint and build blocks.”

This comment illustrates how parents as well as children value the active spending of free time and participation in activities that they recognize as entertaining, educational, and interactive. Most frequently, the participants mentioned shared activities outside of the home (walks in the nature, trips, sports, gardening, holidays, visiting friends or relatives, etc.), and within the household (help with the housework, conversations). Although many of the participants described watching television as their regular activity, it was perceived by most of them as low-quality time and rarely as a social activity that could have a significant impact on the improvement of family relations. This is similar to the findings of some previous studies, although there are also opposite conclusions that watching television can promote interaction to a certain extent and therefore enhance family relationships [5].

Participants’ responses reveal that children and parents succeed in devoting their time to shared activities, mainly on weekends. When asked to describe the family’s typical daily routine on working days and at weekends separately, all families emphasized the importance of non-working days to the quality of family life. In addition to the increased amount of time, some parents also highlighted the value of unstructured time that is not framed by obligations and rigid organization, in terms of both time and content. The following comment of one father supports this: “It seems to me that nowadays as a parent with all the obligations you already have a lot of planning to do and responsibilities, even rules that you have to follow. I take family life as a part of life that may be left more to improvisation, something that is not strictly planned. That we are all relaxed, no one is burdened with work, and we are together, no matter what we do.” Another respondent, a mother of two, also expressed a similar wish: “We spend time together, but there is a lack of quality and unconcerned time. That is, if you really could have time for just sitting on the couch, while you are talking totally carefree. We always have to do something, always in a hurry.”

Although a lack of time during weekdays makes it harder for the participants to spend, what they regard to be, quality time together, they also try to find value in everyday activities, such as a shared drive to work, shopping, cooking, etc. For some, this is the only time they really have together during the week. There are also big differences between the stages in family cycles, since family dynamics of those with pre-school children or adolescents may differ significantly (also in regard to the ICT use). Families with small children emphasized that their everyday life is structured primarily according to the children and their activities. Sometimes, extended family is more involved in everyday activities in such families, since grandparents take a substantial part of the burden for household work and children care, especially if they live in the same house or in the vicinity.

It is often hard for families to engage in activities that would gather all family members. Sometimes this happens because of a lack of time, and sometimes due to age gaps between children or their different interests. However, even in such families, certain events are perceived as an essential part of family life. Namely, all participants expressed the vital importance of sharing daily meal(s) that involve routine and ritual elements [18,32]. In spite of challenging schedules, family members try to gather around the table daily or at least at weekends to discuss different topics, review past events and make new plans; on weekends, this also becomes an opportunity to connect with relocated family members. Almost half of the survey respondents eat a main meal together as a family every day, almost a quarter four times a week, while one-fifth eats together exclusively on weekends. The fact that healthy nutrition habits are important to family members is in consonance with previous researches of various authors [19,20].

To manage challenging schedules, family members from our study mostly use smartphones (avg. 3.03) in contrast to physical tools (avg. 2.20), such as calendars and notepads. Here, the positive attitudes towards the use of technology in everyday life as previously described [14] are clearly observed. Many participants assign the most important role to ICT as a tool for time management and planning; even more often than for assisting in family communication (avg. 3.46). However, it seems that the potential of ICT to impact the life of families remains big, since one of the most common reasons for not spending enough time with other family members is schedule planning inconsistencies (avg. 2.30).

In accordance with this observation, we conclude that the basic functionality of the MyFamily prototype solution (Figure A1) should entail task management, used primarily for logistics and household work coordination in order to promote cooperation and collaboration among family members. To encourage quality family time, it should also introduce some potential recommendations for shared leisure activities.

#### 6.2.3. Healthy Lifestyle

Self-rated health is commonly used when evaluating public health, and researchers are examining associations between ICT and self-rated health among different population groups [33,34]. For the purpose of this study, we asked respondents to assess their lifestyle regarding commonly perceived factors for maintaining and improving health, i.e., their eating habits, physical activity and balance between work, leisure time, and rest. On average (Table 2), the survey respondents perceive themselves as physically active (avg. 3.36), they take time to rest and relax (avg. 3.25), and mostly perceive their eating habits to be healthy (avg. 3.45).

Unhealthy eating habits were exposed by the participants to a much-lesser extent than physical inactivity, despite some other studies on health-related behavior in Slovenia that estimate only a third of the population to have healthy eating habits [35,36]. This perception seems to be partly associated with the availability of school food service and home food gardens. According to an analysis, 46% of the non-farming households in Slovenia were involved in home food production, which is a widespread phenomenon in Slovenia [37].

We were also interested in what our participants perceive as the main obstacles to a healthier lifestyle (Table 3). Most of the respondents complained about three key factors—lack of time (68.7% of respondents), lack of motivation (40.4%) and poor time management (36.4%). Besides, the results also showed that ICT can be regarded as a barrier to healthier habits, since 8.1% of the respondents answered that technology and the way they use it prevent them from engaging in healthier behavior.

In addition, the role of ICT in promoting or maintaining such behavior was clearly less important than in communication and household planning or management. Some respondents use various devices and applications for monitoring physical activity, but they are not widely adopted among our participants, especially compared to time-management applications, e.g., calendar apps. Although the Eurobarometer survey [24] expressed some degree (52%) of demand for e-health solutions, usage reported in our survey is very low. Study participants believe that it cannot, also according to some authors [22], replace face-to-face interaction with health specialists or it poses great privacy risks. Namely, only 13% of women and 16% of men in our survey regularly use mobile applications that help manage their health, such as planning and tracking diet or physical activity or monitoring health indicators. The number of frequent users of time-management apps—reminders or calendars—is almost three times bigger. 

The motivational aspect of mobile health applications was often not clearly perceived by the users. One participant expressed this by saying: “No, it basically does not motivate me, but I see that others are being very motivated. My husband has a lot of these apps, for running etc. Well, I think it is quite often connected with self-praise on social networks. This is not my goal; I do not want to invest time in that. But I am interested in information about me. Let’s say, if I notice my health is getting worse, then I look it up in the app and see it is probably because I was not active enough or did not sleep enough./…/I set goals and try to reach them. For example, if I know I will mostly sit in the office today, then I use my lunch break to go for a walk so that I achieve my daily activity goal.” 

This comment illustrates how tracking behavior can be a valuable source of information for some users. Although perceived by many mainly as a self-monitoring tool, it can have the potential to stimulate changes by setting personal goals and viewing progress constantly. This, however, seems to be limited only to those tools that are not time consuming and do not require a lot of effort from its users. These are the main reasons why some participants stopped using different solutions in the past, and are also in line with our previous findings, which emphasize how time scarcity impacts the choice of a healthy lifestyle as well as the quality and quantity of family time.

For the most-advanced users of the MyFamily prototype solution, we outlined a feature of automatically tracking physical activity (steps) with the existing e-health and fitness tracking mobile application 24@Life connected via data API. Cumulative physical activity of a family can be shown on the application dashboard in order to contribute to a shared experience among family members (Figure A1).

To motivate the usage of activity tracker and higher completion of tasks, which could indicate more solidarity in the family, we propose some extent of gamification mechanisms to be integrated in the prototype solution. It could be presented in the form of collecting experience points for physical activity and participation in family activities. Further usability testing will reveal if these features have the potential to motivate users in this regard.

### 6.3. Limitations of the Study

The constraints of the research were associated with sample selection bias and data analysis, which, though it provided a sufficient basis for the next steps of derivative solution development, may have limited reach in regard to the quality of data exploration and methods used.

The online survey sample was not representative of the general population regarding demographic parameters. In addition, it was conducted among participants that were invited to an online survey via a known email list from a source, which would indicate above-average ICT use, as we were focusing on an explorative, digitally skilled family type that possesses an above-average number of digital devices.

Though semi-structured interviews, a common method in household research, provided us with useful qualitative insight into everyday family ICT use, this method has its disadvantages, such as difficulty to compare and analyze the answers, infer cause and effect, and provide a reliable interpretation without construing too much.

The EkoSmart: Active Life and Well-being Project (RRP3) is based on the iterative solutions development approach. Throughout the whole project timeline, there will be several approaches and exploration techniques tested with different participants and sample sizes that should provide an adequate overall overview of users’ family needs, requirements, situation and practices. Some stages included in our iterative process are: Identifying motivational factors in the use of e-health solutions, determining the concept of a healthy and active family in a smart city, personal creation of typical users, mental models construction, paper prototype testing, and interactive prototype solution usability testing, among others.

Considering available resources, expenses and timeframe, the research presented in this article provides us with enough insights to construct the next steps for the path towards a prototype solution in the form of a progressive web application for everyday family schedule planning that could promote inclusive communication, collaboration and a physically active lifestyle among family members through a simple motivational mechanism. The cause of these constraints can pose a risk that the immediate results of this study will not be effectively relevant for a generalized approach towards the intersection of these topics: ICT use in everyday family life, family time organization and management, and a healthy lifestyle.

## 7. Conclusions

The modern, continually changing world characterized by a flood of information and new modes of communication, sets new challenges that were not present some time ago. Both individuals and social groups often find it difficult to adapt to these new developments, which are demanding in terms of general well-being and health, as well as in terms of interpersonal relationships. In terms of their internal dynamics and their position in the modern world, families are often burdened with the overwhelming use of technology by some members, typically the younger generation, and also underutilization of technology by others, mostly elders, creating a communication barrier within the family. With appropriate innovative ICT solutions, families could be better organized, connected and informed, which could lead to less stress and more enjoyable time for socializing, and at the same time promote a healthy lifestyle at various levels.

Our aim was to develop and test approaches to compose tools for communication, household management and planning, with focus on motivation for a healthier lifestyle and quality family time, as the sense of well-being is closely associated with common family activities and enjoying time together with positive outcomes intended for all generations.

In this paper, we presented an analysis of family members’ behavior regarding ICT use in everyday family life, the organization of family life and time management, and a healthy lifestyle with the use of mixed methodology that combines qualitative and quantitative approaches in the form of an online structured questionnaire survey as well as semi-structured interviews. To capture the greatest extent of practical problems and challenges that occur in everyday family life, we evaluated several forms of extended family with three generations (grandparents, parents, children) as our primary subject of interest.

The study results suggest that children and parents mostly use smartphones, while grandparents mostly use regular cellphones. Parents usually use desktop and laptop computers more than their children. Mothers are most active in household work, whereas fathers are more active in transporting children. Reasons given regarding a lack of quality family time and also a lack of motivation for a healthier lifestyle often involve work overload and insufficient schedule planning by parents, and excess of children’s school and after-school activities. Planning and scheduling of common tasks is typically done via smartphones, not by physical means. Reported interest in mobile health applications and smart health accessories is low or non-existent across all generations.

An improved understanding of family members’ needs, limitations, digital skills and ICT access, everyday activities and attitude towards e-health applications and services provided us with a good sense of requirements regarding the conception of new processes and products that promote a healthy lifestyle through inclusive communication and logistics processes.

We adopted the notion of a family-centered design approach that provides common experience to benefit family members of all generations. A family-centered design is inherently inclusive, paying concern to access to technology among group members, shared purpose and modes of communication when developing ICT solutions. Throughout this approach and study research observations, we outlined a few important prototype solution features: A task management process that allows for different modalities of the application interface (specifically, text messaging service reminders for the unskilled), a progressive web application for the digitally literate, and physical activity tracking for the most advanced users. We also aim to integrate some degree of a motivational gamified mechanism encompassing these functionalities. In continuation of the EkoSmart program: Active Life and Well-Being project (RRP3), we aim to additionally test the results presented in this article for congruence with associated stages of the research and development process.

In accordance with the project, we will apply these findings to future tasks for establishing distinct functionalities of the MyFamily application through the construction of mental models that will be user tested with an interactive prototype solution in the form of a progressive web application. Further, we will connect assorted sensor and user-infused data sources of the solution with an existing e-health and fitness application 24@Life in order to achieve some degree of integration into a smart city ecosystem.

## Figures and Tables

**Table 1:** Communication devices most commonly used by different family members.

Family role	Smartphone	Computer	Mobile phone	Tablet
Mothers	80.4%	81.4%	29.9%	34%
Fathers	83.2%	83.2%	24.2%	34.7%
Children	80.2%	70.4%	21%	43.2%
Grandmothers	31.8%	34.1%	63.6%	12.5%
Grandfathers	27.6%	40.8%	63.2%	13.2%

**Table 2:** Health activities self-assessment (scale: 1—disagree, never; 5—agree, often).

Assertion	Mean	Std. Dev.
I am often physically active	3.36	1.106
I often take time for rest and relaxation	3.25	1.009
I consider my eating habits to be healthy	3.45	0.985

**Table 3:** Obstacles to a healthier lifestyle, multiple answers.

Obstacles	Frequency %
Lack of time	68.7%
Lack of motivation	40.4%
Poor time management	36.4%
Use of ICT	8.1%
Other	1%
